# Gallstone Disease and the Risk of Type 2 Diabetes

**DOI:** 10.1038/s41598-017-14801-2

**Published:** 2017-11-20

**Authors:** Jun Lv, Canqing Yu, Yu Guo, Zheng Bian, Ling Yang, Yiping Chen, Shanpeng Li, Yuelong Huang, Yan Fu, Pan He, Aiyu Tang, Junshi Chen, Zhengming Chen, Lu Qi, Liming Li

**Affiliations:** 1 Department of Epidemiology and Biostatistics, School of Public Health, Peking University Health Science Center, Beijing, China; 2 Peking University Institute of Environmental Medicine, Beijing, China; 3 Chinese Academy of Medical Sciences, Beijing, China; 4 Clinical Trial Service Unit & Epidemiological Studies Unit (CTSU), Nuffield Department of Population Health, University of Oxford, Oxford, United Kingdom; 5 Qingdao Center for Disease Control & Prevention, Qingdao, Shandong China; 6 Hunan Center for Disease Control & Prevention, Changsha, Hunan China; 7 Hainan Center for Disease Control & Prevention, Haikou, Hainan China; 8 Huixian Center for Disease Control & Prevention, Huixian, Henan China; 9 Suzhou Center for Disease Control & Prevention, Suzhou, Jiangsu China; 10 China National Center for Food Safety Risk Assessment, Beijing, China; 11 Department of Epidemiology, School of Public Health and Tropical Medicine, Tulane University, New Orleans, Louisiana United States of America; 12 Department of Nutrition, Harvard School of Public Health, Boston, Massachusetts, United States of America

## Abstract

Gallstone disease (GSD) is related to several diabetes risk factors. The present study was to examine whether GSD was independently associated with type 2 diabetes in the China Kadoorie Biobank study. After excluding participants with prevalent diabetes and prior histories of cancer, heart disease, and stroke at baseline, 189,154 men and 272,059 women aged 30–79 years were eligible for analysis. The baseline prevalence of GSD was 5.7% of the included participants. During 4,138,687 person-years of follow-up (median, 9.1 years), a total of 4,735 men and 7,747 women were documented with incident type 2 diabetes. Compared with participants without GSD at baseline, the multivariate-adjusted hazard ratios (HRs) for type 2 diabetes for those with GSD were 1.09 (95% CI: 0.96–1.24; P = 0.206), 1.21 (95% CI: 1.13-1.30; P < 0.001), and 1.17 (95% CI: 1.10-1.25; P < 0.001) in men, women, and the whole cohort respectively. There was no statistically significant heterogeneity between men and women (*P* = 0.347 for interaction). The association between GSD and type 2 diabetes was strongest among participants who reported ≥5 years since the first diagnosis and were still on treatment at baseline (HR = 1.48; 95% CI: 1.16-1.88; P = 0.001). The present study highlights the importance of developing a novel prevention strategy to mitigate type 2 diabetes through improvement of gastrointestinal health.

## Introduction

Type 2 diabetes has become epidemic worldwide^1^. In China, a rapid increase in diabetes incidence was observed in recent decades, with a prevalence of 11.6% in 2010^2^. Gallstone disease (GSD) remains a common gastrointestinal disorder in both developed countries^3^ and Asian populations such as Chinese^4,5^. GSD is related to several cardiometabolic risk factors such as obesity, dyslipidemias (hypertriglyceridemia and low high-density lipoprotein cholesterol), unhealthy diet, and sedentary lifestyle^3,6^. Several prospective studies have supported that the presence of GSD was associated with increased risk of coronary heart disease, which could not be explained by traditional risk factors^7^. Whether there is a similar association between GSD and type 2 diabetes remains unclear. Previous studies which have related GSD to diabetes were limited by cross-sectional design^8–10^. To our knowledge, only one prospective study on the relation between GSD and increased risk of type 2 diabetes has been reported^11^.

We have previously shown an association between GSD and ischemic heart disease in a large prospective cohort of 0.5 million adults − the China Kadoorie Biobank (CKB) study^12^. We aimed to examine the association between GSD and the risk of incident type 2 diabetes in the same population. We also assessed potential interactions between GSD and conventional risk factors for type 2 diabetes.

## Results

### Baseline characteristics of study participants

At baseline, 5.7% of the 461,213 participants reported the presence of GSD (men, 3.6%; women, 7.1%). Compared with participants without GSD, those with GSD were older, more likely to be urban residents, had higher body mass index (BMI) and waist circumference (WC) values, had a higher prevalence of chronic hepatitis/cirrhosis and peptic ulcer, and had a higher weight increase since 25 years of age (Table 1). Women with GSD had an earlier age at the first diagnosis and longer duration than men with GSD (P < 0.001).

**Table 1:** Baseline characteristics of the 461,213 participants according to the presence of gallstone disease.

Baseline characteristics	Men			Women		
	With GSD	Without GSD	*P* Value	With GSD	Without GSD	*P* Value
No. of participants	6,854	182,300	—	19,353	252,706	—
Age (year)	53.5	51.5	<0.001	52.9	49.9	<0.001
Urban area (%)	54.5	41.0	<0.001	44.8	42.6	<0.001
Currently married (%)	94.5	92.9	<0.001	90.2	89.7	0.033
Middle school and above (%)	62.2	57.4	<0.001	47.0	43.5	<0.001
Current daily smoker (%)	65.5	68.2	<0.001	2.8	2.6	0.210
Current daily drinker (%)	15.7	21.3	<0.001	0.7	1.0	<0.001
Physical activity (MET-hour/day)	21.4	23.0	<0.001	20.2	21.2	<0.001
Average weekly consumption^a^						
Red meat (day)	4.0	4.0	0.228	3.5	3.5	0.056
Fresh vegetables (day)	6.9	6.8	0.105	6.84	6.83	0.032
Fresh fruits (day)	2.5	2.2	<0.001	3.0	2.8	<0.001
BMI (kg/m^2^)	23.8	23.3	<0.001	24.1	23.6	<0.001
WC (cm)	83.2	81.5	<0.001	79.7	78.4	<0.001
Prevalence of						
Hypertension (%)	33.0	34.9	<0.001	29.5	30.7	<0.001
Chronic hepatitis/cirrhosis (%)	4.3	1.6	<0.001	1.4	0.8	<0.001
Peptic ulcer (%)	8.6	5.2	<0.001	5.4	2.7	<0.001
Weight change since 25 years (kg)^b^	5.7	4.5	<0.001	5.7	4.7	<0.001
Weight change during the past 12 months (%)^c^						
Same as before	76.5	79.7	—	73.8	77.8	—
Gain of ≥2.5 kg	10.2	10.2	0.342	13.8	12.3	<0.001
Loss of ≥2.5 kg	13.3	10.1	<0.001	12.4	9.9	<0.001
Postmenopausal (%)	—	—	—	50.1	49.1	<0.001
Family history of diabetes (%)	7.9	5.8	<0.001	8.0	6.4	<0.001
Characteristics of GSD^d^						
Age at the first diagnosis (year)	44.9	—	—	43.9	—	<0.001^e^
Duration since the first diagnosis (year)	8.1	—	—	9.2	—	<0.001^e^
Still on treatment at baseline	13.0	—	—	14.6	—	0.001^e^

GSD indicates gallstone disease; MET, metabolic equivalent task; BMI, body mass index; and WC, waist circumference.

The results are presented as adjusted means or percentages. All variables are adjusted for age and survey sites, as appropriate.

^a^The average weekly consumptions of red meat, fresh vegetables, and fruits were calculated by assigning participants the midpoint of their consumption category.

^b^74,458 participants with a missing value for self-reported weight at 25 years of age were excluded from this analysis.

^c^Multinomial logistic regression was used for testing, and the “same as before” was used as the reference category.

^d^There were statistically significant differences in all three characteristics (*P* ≤ 0.001) between men and women.

^e^P value for comparison between men and women.

### Association between GSD and incident type 2 diabetes

During a median follow-up of 9.1 years (interquartile range: 1.92 years; total person-years: 4,138,687), there were 4,735 incident cases of type 2 diabetes in men and 7,747 in women. In age- and sex-adjusted (model 1) and multivariable-adjusted analyses in the whole cohort (model 2), the presence of GSD was associated with increased risk of incident type 2 diabetes (Table 2). The association was moderately attenuated after further adjustment for BMI and WC (model 3). Compared with participants without GSD at baseline, the adjusted hazard ratio (HR) for type 2 diabetes (model 3) was 1.17 (95% confidence interval [CI]: 1.10−1.25; P < 0.001) for those with GSD in the whole cohort. There was no statistically significant heterogeneity between men (HR = 1.09; 95% CI: 0.96−1.24; P = 0.206) and women (HR = 1.21; 95% CI: 1.13−1.30; P < 0.001) in the aforementioned association (*P* = 0.347 for interaction with sex). These associations were not materially changed with additional adjustment for weight change since 25 years of age; or additional adjustment for significant weight change during the past 12 months; or additional adjustment for histories of chronic hepatitis/cirrhosis and peptic ulcer; or excluding participants with type 2 diabetes occurring during the first two years of follow-up (data not shown).

**Table 2:** HRs (95% CIs) for the association between gallstone disease and incident type 2 diabetes.

	Person-years	Cases	Age- and sex-adjusted (model 1)	Multivariable-adjusted^a^ (model 2)	Further adjustment for BMI and WC (model 3)
Total					
Without GSD	3,905,602	11,352	1.00	1.00	1.00
With GSD	233,084	1,130	1.33 (1.25−1.42)	1.32 (1.24−1.40)	1.17 (1.10−1.25)
Men					
Without GSD	1,615,585	4,484	1.00	1.00	1.00
With GSD	59,953	251	1.29 (1.13−1.46)	1.22 (1.08−1.39)	1.09 (0.96−1.24)
Women					
Without GSD	2,290,017	6,868	1.00	1.00	1.00
With GSD	173,131	879	1.33 (1.24−1.43)	1.33 (1.24−1.43)	1.21 (1.13−1.30)

HR indicates hazard ratio; CI, confidence interval; GSD, gallstone disease; BMI, body mass index, and WC, waist circumference.

^a^Adjusted for age (years), sex (for the whole cohort), level of education (no formal school, primary school, middle school, high school, college, or university or above), marital status (married, widowed, divorced/separated, or never married), alcohol consumption (never, former, current weekly, current daily <15, 15–29, 30–59, or ≥60 g per day), smoking status (never, former, current daily <15, 15–24, or ≥25 cigarettes or equivalents per day; former smokers who stopped smoking for illness were included in the current smoker category to avoid misleadingly elevated risk), level of physical activity (MET-hours/day), intake frequencies of red meat, fresh fruits, and vegetables (daily, 4–6 days/week, 1–3 days/week, monthly, or rarely or never), prevalent hypertension (yes or no), family history of diabetes (yes or no), and menopausal status (premenopausal, perimenopausal, or postmenopausal; for women only).

### Stratified analysis

We performed stratified analysis according to the combined categories of duration of GSD from the first diagnosis to the baseline and treatment status at baseline. The association of GSD with type 2 diabetes appeared to be strongest among those who reported more than five years of duration and were still on treatment at baseline, with HR of 1.48 (95% CI: 1.16−1.88; P = 0.001) in the whole cohort (Table 3).

**Table 3:** HRs (95% CIs) for the association between gallstone disease and incident type 2 diabetes according to the combined categories of duration since the first diagnosis and treatment status at baseline.

	Person-years	Cases	HRs (95% CIs)
Total			
≤5 years, no treatment	95,516	504	1.25 (1.14−1.37)
≤5 years, still on treatment	20,326	82	1.21 (0.97−1.51)
>5 years, no treatment	103,934	475	1.06 (0.96−1.16)
>5 years, still on treatment	13,057	68	1.48 (1.16−1.88)
Men			
≤5 years, no treatment	27,077	121	1.13 (0.94−1.35)
≤5 years, still on treatment	4,966	19	1.13 (0.72−1.78)
>5 years, no treatment	25,307	98	0.99 (0.81−1.21)
>5 years, still on treatment	2,584	13	1.40 (0.81−2.42)
Women			
≤5 years, no treatment	68,439	383	1.31 (1.18−1.45)
≤5 years, still on treatment	15,360	63	1.25 (0.97−1.60)
>5 years, no treatment	78,627	377	1.08 (0.97−1.20)
>5 years, still on treatment	10,473	55	1.50 (1.15−1.96)

HR indicates hazard ratio; and CI, confidence interval.

The reference group was participants without GSD at baseline. Multivariable model was adjusted for age (years), sex (for the whole cohort), level of education (no formal school, primary school, middle school, high school, college, or university or above), marital status (married, widowed, divorced/separated, or never married), alcohol consumption (never, former, current weekly, current daily <15, 15–29, 30–59, or ≥60 g per day), smoking status (never, former, current daily <15, 15–24, or ≥25 cigarettes or equivalents per day; former smokers who stopped smoking for illness were included in the current smoker category to avoid misleadingly elevated risk), level of physical activity (MET-hours/day), intake frequencies of red meat, fresh fruits, and vegetables (daily, 4–6 days/week, 1–3 days/week, monthly, or rarely or never), prevalent hypertension (yes or no), family history of diabetes (yes or no), menopausal status (premenopausal, perimenopausal, or postmenopausal; for women only), body-mass index (kg/m^2^), and waist circumference (cm).

**Figure 1:**
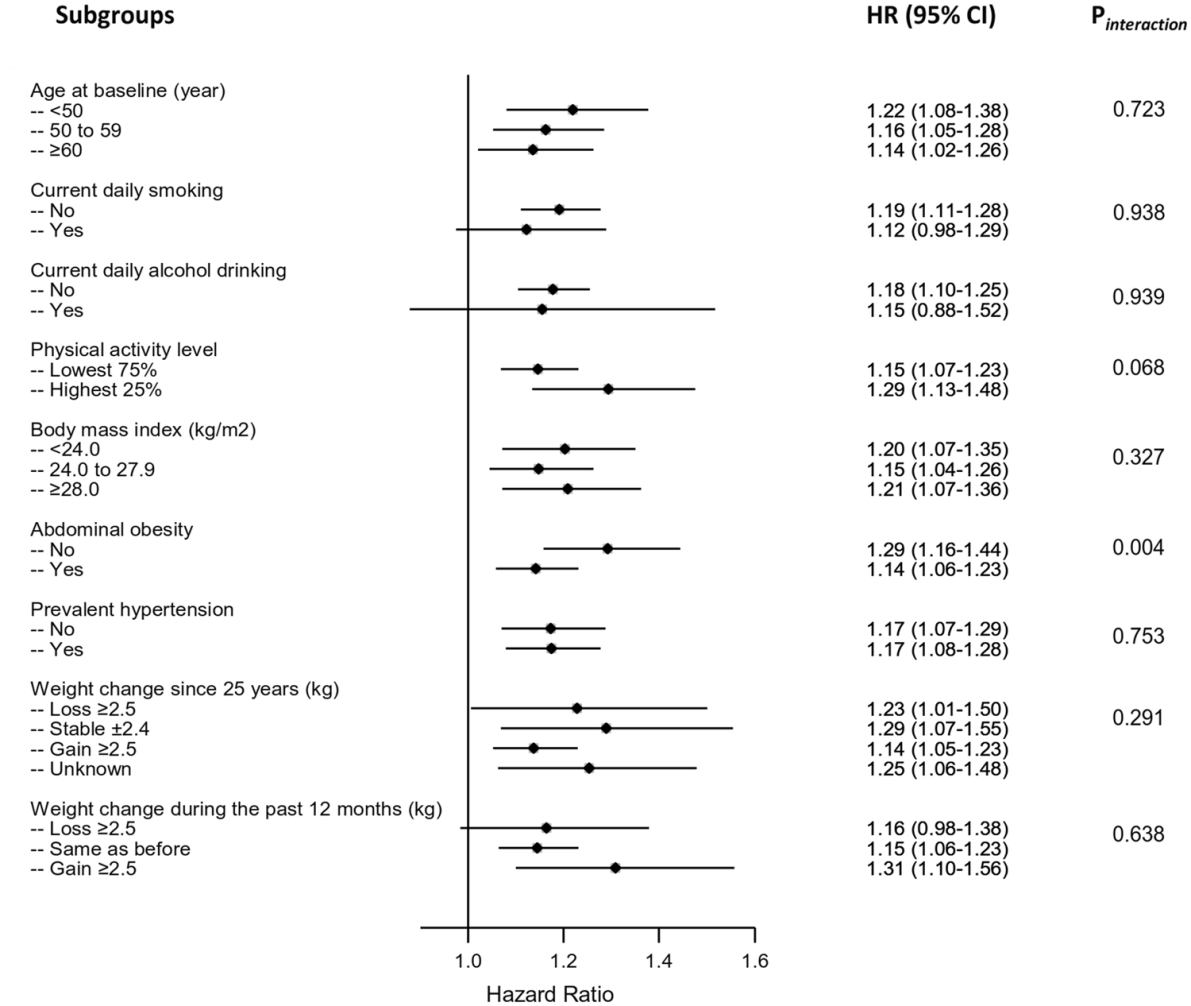
Subgroup analyses of the association between gallstone disease and type 2 diabetes according to potential baseline risk factors. Adjustments were made for age, sex, education, marital status, alcohol consumption, smoking status, physical activity, intakes of red meat, fresh fruits, and vegetables, prevalent hypertension, family history of diabetes, body-mass index, and waist circumference.

We also analyzed the association between GSD and type 2 diabetes according to other potential baseline risk factors. The positive associations were similar across subgroups stratified according to age, smoking status, alcohol consumption, level of physical activity, BMI, prevalent hypertension, weight change since 25 years of age, and weight change during the past 12 months in the whole cohort (Fig. 1) and in both men and women (data not shown) (all *P* values for interaction >0.05). Notably, statistically significant difference was observed across strata by the presence of abdominal obesity defined by WC in the whole cohort (P = 0.004 for interaction) and women (P = 0.018 for interaction), but not in men (P = 0.455 for interaction). The positive association between GSD and type 2 diabetes was stronger in non-abdominal obese (HR, 95% CI: 1.29, 1.16−1.44 for the whole cohort; 1.35, 1.19−1.54 for women) than abdominal obese participants (HR, 95% CI: 1.14, 1.06−1.23 for the whole cohort; 1.16, 1.06−1.27 for women). The corresponding HRs (95% CIs) for men was 1.13 (0.90−1.41) in non-abdominal obese participants and 1.09 (0.93−1.27) in abdominal obese participants.

## Discussion

In this large prospective study with close to ten years of follow-up, we observed that the presence of GSD was prospectively associated with increased risk of type 2 diabetes after adjustment for potential confounding from traditional risk factors of type 2 diabetes. Such association was strongest among participants who had a long history of GSD and were still on treatment at baseline. The association between GSD and type 2 diabetes was consistent in men and women, but the stronger association was observed in non-abdominal obese than in abdominal obese women.

To our knowledge, only one study has prospectively examined the association of GSD with type 2 diabetes in a European population (EPIC-Potsdam study) with a mean follow-up of 7.0 years^11^. It was consistent with our findings that persons with GSD had an increased risk of type 2 diabetes (HR = 1.42; 95% CI: 1.21–1.68) after adjustment for sex, age, WC, and lifestyle risk factors.

Several potential mechanisms may help explain the association between GSD and type 2 diabetes. Higher prevalence of GSD has been reported in persons with obesity^13,14^, hyperinsulinemia^8,15^, insulin resistance^16^, and metabolic syndrome^17^. The coexistence of these risk factors for type 2 diabetes might be the reason that participants with GSD had an increased diabetes risk. In the current study, the adjustment for BMI and WC moderately attenuated the association between GSD and type 2 diabetes, suggesting that obesity might only partly explain the higher risk of type 2 diabetes in patients with GSD. In addition, statistically significant associations remained after adjustment for risk factors such as hypertension and lifestyle factors, suggesting other mechanisms might also be involved. A similar change in the risk estimates with adjustment for potential confounders was also observed in the EPIC-Potsdam study^11^. In addition, we explored whether the association between GSD and type 2 diabetes was confounded by long- and short-term weight change, which has been related to both type 2 diabetes^18,19^ and gallstone formation^3^. Additional adjustment for weight change since 25 years of age or significant weight change in the past 12 months did not affect the association appreciably.

Recent studies in both human^20^ and experimental animals^21^ have linked gut microbiota dysbiosis with the formation of cholesterol gallstones. This relation is probably through distorted secretion of bile acids because the bile acids play a key role in regulating abundance or metabolism of gut microbiota^22^. Accumulating evidence implicates the involvement of gut microbiota in the development of type 2 diabetes and cardiovascular diseases^23,24^. The associations of GSD with type 2 diabetes and ischemic heart disease^12^ we observed in the same population suggest that the increased risks associated with GSD might be at least partly through affecting gut microbiota metabolism. Also, both of the associations were independent of each other because we excluded participants with a history of heart disease (or diabetes) at baseline for the analysis of diabetes (or ischemic heart disease^12^). Our findings would motivate further investigation of this hypothesis.

In the present study, the association between GSD and type 2 diabetes was dependent on the abdominal obese status in women. The stronger association was observed in non-abdominal obese than abdominal obese women. Weikert *et al*. reported a similar interaction between GSD and abdominal obesity on type 2 diabetes^11^. It is possible that obese women already had a high risk of diabetes, and GSD added only modestly deleterious effect on the relative scale. However, the absolute risk associated with abdominal obesity among women with GSD was much greater than those without abdominal obesity.

To our knowledge, this is one of the largest prospective studies that examined the association between GSD and type 2 diabetes. We carefully adjusted for potential confounders. We excluded participants with type 2 diabetes at baseline and those with type 2 diabetes occurring during the first two years of follow-up to minimize the potential bias arising from reverse causality.

However, several limitations warrant mention. First, the presence of GSD was self-reported. The possibility of including participants with asymptomatic GSD in the non-GSD group could lead to misclassification, which was more likely to be non-differential in a prospective study design. Second, the present study lacked information on the subtypes of gallstones. However, most gallstones in the Chinese population have been of the cholesterol type since the early 1990s^25^. We did not ask the severity of GSD, such as asymptomatic, symptomatic, or having undergone cholecystectomy, limiting our in-depth analysis. Third, residual confounding by other factors such as more detailed dietary factor, dyslipidemia, and hyperinsulinemia remains possible. However, Weikert *et al*. reported that the association between GSD and the risk of type 2 diabetes was not substantially attenuated after further adjustment for selected biomarkers including glucose, total cholesterol, and triglycerides^11^. Fourth, baseline identification of prevalent diabetes relied on the self-reported previous clinical diagnosis or on-site glucose testing, and identification of incident diabetes during follow-up relied mainly on the local disease and death registries and health insurance system. Missing some asymptomatic diabetes cases was inevitable. However, such misclassification was more likely to be nondifferential and might lead to attenuation of effect estimates.

Our findings showed an increased risk of incident type 2 diabetes associated with the presence of GSD. It highlights the importance of developing a novel prevention strategy to mitigate type 2 diabetes through improvement of gastrointestinal health (e.g., timely treatment of gastrointestinal diseases and maintaining healthy gut microbiota). More studies are warranted to confirm the association and to elucidate the potential biological mechanisms.

## Methods

### Study population

Details of the CKB study is available elsewhere^26,27^. Briefly, we enrolled 512,891 adults aged 30−79 years from 10 geographically diverse localities across China during 2004–08. All participants eligible for subsequent analysis had completed questionnaire, physical measurements, and a written informed consent form. The CKB study was approved by the Ethical Review Committee of the Chinese Center for Disease Control and Prevention (Beijing, China) and the Oxford Tropical Research Ethics Committee, University of Oxford (UK). All methods were performed in accordance with relevant guidelines and regulations.

For our analyses, we excluded 30,300 participants who had self-reported diabetes or screen-detected diabetes at baseline. Diabetes detected by on-site screening was defined as a fasting blood glucose ≥7.0 mmol/L or a random blood glucose ≥11.1 mmol/L^28^. We also excluded those who reported prior medical histories of cancer (*n* = 2,577), heart disease (*n* = 15,472), and stroke (*n* = 8,884), and those who had incomplete data of BMI (*n* = 2). We finally included 189,154 men and 272,059 women in the analysis.

### Assessment of exposure

At baseline survey, trained staff administered a standardized questionnaire using a laptop-based data-entry system, with built-in functions to prevent logical errors and missing items. Participants reported whether they had ever been diagnosed with GSD (yes or no), with or without cholecystitis complication, by a doctor, the age of their first diagnosis, and whether they were still on treatment (yes or no).

### Assessment of covariates

Trained staff collected socio-demographic characteristics (age, sex, education, and marital status), lifestyle behaviors (tobacco smoking, alcohol consumption, physical activity, and intakes of red meat, fresh fruits, and vegetables), personal health and medical history (hypertension, chronic hepatitis/cirrhosis, peptic ulcer, body weight at 25 years of age, and significant weight change during the past 12 months), women’s menopausal status, and family medical history. Questions about alcohol consumption used in the present analyses included drinking frequency, alcoholic beverage type, and volume of alcohol drunk on a typical drinking day in the past year. Questions about smoking included frequency, type, and amount of tobacco smoked per day for ever smokers, and the reason for quitting for former smokers. Questions about physical activity included type and duration of activities in occupational, commuting, domestic, and leisure-time related domains in the past 12 months. Habitual dietary intake in the past year was assessed by a qualitative food frequency questionnaire.

At baseline, body weight, height, WC, and blood pressure were measured by trained staff using calibrated instruments. Weight change since 25 years of age was calculated by subtracting recalled weight at 25 years from measured weight at baseline. BMI was calculated as weight in kilograms divided by height in meters squared.

### Ascertainment of incident type 2 diabetes

Incident cases of type 2 diabetes were identified by linking to local disease and death registries, to the national health insurance system, and by active follow-up^27^. The 10^th^ revision of the International Classification of Diseases (ICD-10) was used to code all incident cases of type 2 diabetes by trained staff members who were “blinded” to baseline information. In the present study, we included diabetes cases coded as E11 and E14. Other cases clearly defined as non-type 2 diabetes were excluded. Because most participants in the present study were aged over 40 years among whom the number of any non-type 2 diabetes was small, misclassification of other types of diabetes was minimal.

The validity of outcome ascertainment was verified in a subsample of 831 CKB participants who were identified with the incidence of nonfatal type 2 diabetes during 2004–08 and whose medical records were retrieved. All medical records were reviewed, and diagnoses were adjudicated by clinical research fellows in the Oxford International Coordinating Centre of the CKB in 2012. Of 831 cases, the diagnosis of type 2 diabetes was confirmed in 819 (98.6%).

### Statistical analyses

We compared baseline characteristics between participants with and without GSD using analysis of covariance for continuous variables, and binary or multinomial logistic regression for categorical variables, with adjustment for age and survey sites. We calculated person-years at risk from the baseline recruitment date to the diagnosis of type 2 diabetes, death, loss to follow-up, or December 31, 2015, whichever came first. We used Cox proportional hazards model to estimate the HRs and the 95% CIs, with age as the underlying time scale, and stratified jointly by survey site and age at baseline in 5-year interval.

The multivariable model 1 was adjusted for age and sex. Model 2 additionally adjusted for education, marital status, smoking status, alcohol consumption, physical activity, intake frequencies of red meat, fresh fruits, and vegetables, prevalent hypertension at baseline, family history of diabetes, and menopausal status. Model 3 further included BMI and WC in the model 2. We performed four sensitivity analyses on the basis of model 3: (1) additionally adjusting for weight change since 25 years of age (loss of ≥5.0 kg, loss of 2.5−4.9 kg, stable ± 2.4 kg, gain of 2.5−4.9 kg, gain of 5.0−9.9 kg, gain of 10.0−14.9 kg, gain of ≥15.0 kg, or unknown due to missing information about weight at 25 years of age); (2) additionally adjusting for weight change during the past 12 months (same as before, gain of ≥2.5 kg, or loss of ≥2.5 kg); (3) additionally adjusting for the histories of digestive system diseases including chronic hepatitis/cirrhosis and peptic ulcer; and (4) excluding participants with type 2 diabetes occurring during the first two years of follow-up.

Subgroup analysis was conducted among combined categories of duration since their first diagnosis of GSD and treatment status at baseline, all compared with those without GSD at baseline. We also examined the association between GSD and type 2 diabetes across several baseline subgroups: age, smoking status, alcohol consumption, level of physical activity, BMI, abdominal obesity (defined as WC ≥85 cm in men and ≥80 cm in women), prevalent hypertension, weight change since 25 years of age, and weight change during the past 12 months. Interactions were tested using likelihood-ratio tests comparing models with and without cross-product terms between the baseline stratifying variable and GSD.

We performed all statistical analyses with Stata (version 14.2, StataCorp, College Station, TX, USA). All *P* values were two-sided, and statistical significance was defined as *P* < 0.05.

### Data availability

The datasets analyzed during the current study are available from the corresponding author on reasonable request.
